# The HLA-Cw12 Allele Is an Important Susceptibility Allele for Psoriasis and Is Associated with Resistant Psoriasis in the Turkish Population

**DOI:** 10.1155/2019/7848314

**Published:** 2019-06-25

**Authors:** Nahide Onsun, Serpil Pirmit, Dilek Ozkaya, Şirin Çelik, Aylin Rezvani, F. Pelin Cengiz, Cigdem Kekik

**Affiliations:** ^1^Department of Dermatology, Bezmialem Vakif University Medical School, Turkey; ^2^Istanbul Lepra Hospital, Ministry of Health, Turkey; ^3^Deparment of Rheumatology, Bezmialem Vakif University Medical School, Turkey; ^4^Istanbul University Medical School Medical Biology Department, Turkey

## Abstract

**Background:**

Psoriasis is a multifactorial immune-mediated inflammatory disease triggered by both genetic and environmental factors. The strong association between psoriasis and HLA-C⁎06 allele has been demonstrated in various races. The HLA-C⁎12 allele is closely related to the HLA-C⁎06 family of alleles and shares identical sequences. To the best of our knowledge, there is no information about the relationship between HLA-C⁎12 and psoriasis in the Turkish population. The present study aims to determine this relationship.

**Methods:**

This case control study involved 150 patients with plaque-type psoriasis and 145 age- and gender-matched healthy individuals. Severity of psoriasis was measured using the PASI scores of all patients and joint involvement was investigated with CASPAR criteria. HLA-C alleles were determined with a Tepnel-Lifecodes system.

**Results:**

HLA-C⁎06, HLA-C⁎12, and HLA-C⁎04 alleles were most commonly observed in psoriasis patients. HLA-C⁎06 and HLA-C⁎12 were significantly more frequent in the psoriasis group. HLA-C⁎06 was 4.11 times more common in psoriasis patients. An increase in PASI (Psoriasis Area Severity Index) scores was compatible with HLA-C⁎12 positivity. A need for systemic treatment was highly noticeable in patients with the HLA-C⁎12 allele.

**Conclusions:**

HLA-C⁎12 was found as the second most frequent allele with psoriasis in Turkish population and was associated with severe psoriasis. Our study is limited as we could not investigate other potentially related alleles other than HLA-C alleles and risk factors increasing severity of psoriasis.

## 1. Introduction

Psoriasis is a chronic inflammatory disease characterized by T-cell-mediated hyperproliferation of keratinocytes. Both environmental and genetic factors are involved in the aetiology of psoriasis [1]. The HLA-C locus on chromosome 6p21.33 is accepted as a susceptibility locus for psoriasis. The association between psoriasis and the HLA-C*∗*06 allele has been demonstrated in various genetic studies [2–5]. Early-onset psoriasis is more likely to be familial and associated with HLA-C*∗*06, according to the results of population studies [6].

The HLA-C*∗*12 family of alleles is closely related to HLA-C*∗*06 alleles, sharing identical sequences in their alpha-2 domains, peptide binding pockets A, D, E, and all 3 introns [7]. HLA-C*∗*12 has also been found to be a risk factor for psoriatic arthritis [8]. Large epidemiological studies are lacking in Turkey. In a study done with limited number of patients the prevalence was found as 1.3% [8]. Since there are limited numbers of studies that have been made in Turkey for genetic susceptibility factors in patients with psoriasis, we aimed to investigate frequency of HLA-C alleles in the Turkish population.

## 2. Patients and Methods

The study was approved by the Local Ethics Committee of Bezmialem Vakıf University, and written informed consent was obtained from all subjects. One hundred fifty patients (85 men and 65 women) with plaque-type psoriasis were recruited consecutively from the outpatient psoriasis clinic of our dermatology department. The control group consisted of 145 healthy donors who admitted to the Research Centre of Istanbul University (DETAM) for transplantation. The HLA identifications of the patient and control groups were performed in the Medical Biology Department of Istanbul University.

## 3. Genotyping for HLC Alleles

DNA from patient and control groups were isolated from peripheral blood collected into tubes with EDTA by using the “Precipitation with Sodium Chloride Salts” method. DNA samples which would be characterized were prepared for amplification with HLA-C kits (Tepnel Lifecodes). For each HLA characterization, a mix of mastermix, distilled water, and Taq polymerase enzyme was prepared and was completed with DNA. After this procedure, amplification was performed in a cycler (Perkin Elmer). Following the amplification process, 5 ml of samples was coated on plates (Costar). Then, samples were heated at 55°C for 7 minutes in a sonicator (Ultrasonic Cleaner) and were left to wait for 15 seconds followed by an addition of 15 ml of vortexed beads coming from the HLA-C kits. Then, samples were reloaded into the cycler device for the hybridization process. Towards the end of the cycle, 170 *μ*L of a dilution+Streptavidin mix was added onto the samples and samples were loaded into a Luminex device (Lifematch fluoroanalyzer device, Tepnel Lifecodes). Assessments of samples were conducted automatically by the system.

The genotyping system identified 13 alleles in the HLA-C loci: HLA-C*∗*01, HLA-C*∗*02, HLA-C*∗*03, HLA-C*∗*04, HLA-C*∗*05, HLA-C*∗*06, HLA-C*∗*07, HLA-C*∗*08, HLA-C*∗*12, HLA-C*∗*14, HLA-C*∗*15, HLA-C*∗*16, and HLA-C*∗*17. The frequency of each HLA-C allele was evaluated, and the most frequent alleles were determined in both groups.

Severity of psoriasis was measured with the Psoriasis Area and Severity Index (PASI) by two experienced dermatologists, and the mean was taken. The severity of psoriasis was regarded as mild in patients with a PASI score between 1 and 5; moderate in those with a PASI score between 5 and 10; and severe in those with a PASI score of 10 or higher.

All patients with joint symptoms were examined by an experienced rheumatologist. Psoriatic arthritis diagnosis was made according to the CASPAR criteria.

## 4. Statistical Analysis

Categorical variables were summarized as counts and percentages; continuous variables were summarized as means, standard deviations, medians, and minimum and maximum values.

Categorical variables were compared using chi square or Fisher's exact tests, where appropriate. Continuous variables were compared with the Student t-test or Mann-Whitney U, depending on the distribution pattern of the data. The estimation of the effects of several parameters on the presence of specific HLA-C alleles was evaluated with univariate logistic regression models. Results were summarized by using odds ratios, 95% confidence intervals of odds ratios, and p values. Odds ratios for psoriatic arthritis and PASI > 10 were calculated by SPSS.

The overall significance level of the analyses was set to 0.05. Licensed SPSS version 21.0 was used for analysis.

## 5. Results

**Figure 1:**
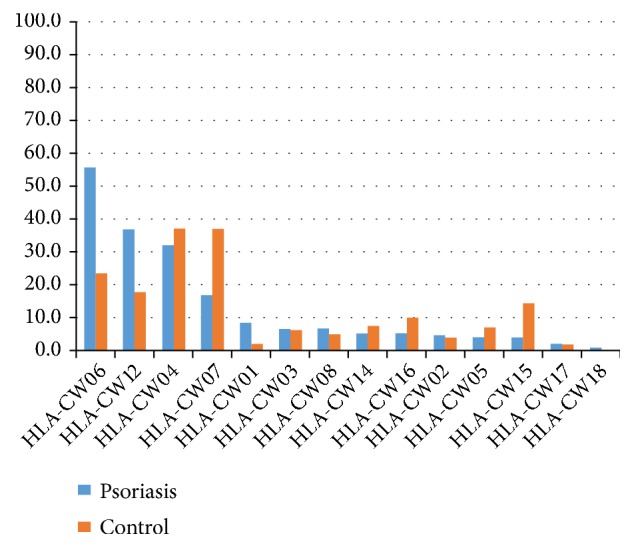
HLA-C allele frequencies in psoriasis and control groups.

HLA-C allele frequencies of psoriasis patients and those of the control group are shown in Figure 1. HLA-C*∗*06 (n=83, 55.7%), HLA-C*∗*12 (n=53, 35%), and HLA-C*∗*04 (n=48, 32%) were the most commonly identified alleles in the psoriasis group. The most frequent alleles in the control group were HLA-C*∗*04 (n=54, 37.2%) and HLA-C*∗*07 (n=54, 37.2%). There were significant differences between the psoriasis and control groups (Figure 1). HLA-C*∗*01, HLA-C*∗*06, and HLA-C*∗*12 alleles were significantly frequent in psoriasis patients compared to controls (8.1 % vs 2.1%; 55.7 % vs 23.4 %; 36.9 % vs 17.9; P= 0.02; P= < 0.001; P= < 0.001). HLA-C*∗*07 and HLA-C*∗*15 were more frequent in control group rather than psoriasis group (37.2 % vs 16.8 %; 14.5 % vs 4%; P= < 0.001; P= 0.002). There were no statistical significant differences related to other alleles between psoriasis and patient groups.

**Figure 2:**
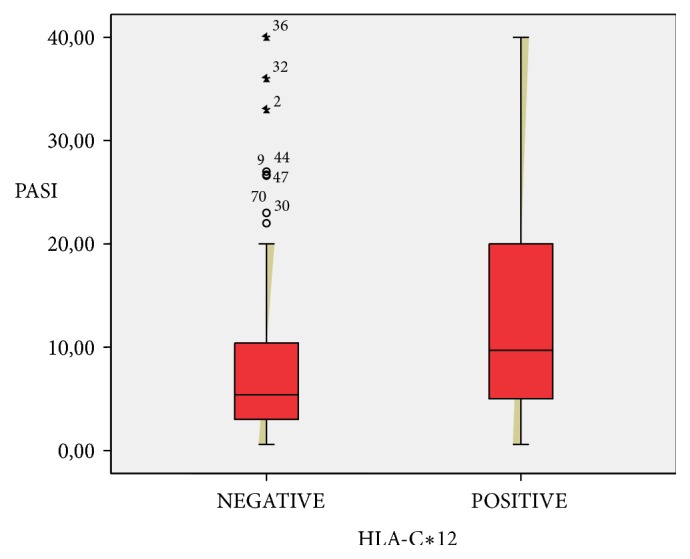
Association of high PASI scores with HLA-C*∗*12.

The strong association between psoriasis and HLA-C*∗*06 allele was confirmed in the Turkish population. HLA-C*∗*06 positivity was present in 55.7 % of patient group. HLA-C*∗*06 increases the risk of psoriasis by 4.11 times, whilst carrying the HLA-C*∗*12 allele increases the risk by 2.68 times. An association between HLA-C*∗*06 and early onset of psoriasis was also observed (Table 1). Furthermore, there was a significant association between HLA-C*∗*12 and the Psoriasis Area Severity Index (PASI). The mean PASI score of the 53 patients with HLA-C*∗*12-positive was 12.8, while the mean PASI score of HLA-C*∗*12-negative patients was 8.39 (Tables 2, 3, and 4 and Figure 2). The difference was statistically significant (P= 0.011).

Almost all psoriasis patients carrying the HLA-C*∗*12 allele had PASI scores over 10 and they were resistant to topical and conventional systemic treatments and phototherapy (Table 5). It seems that HLA-C*∗*12 increases severity of psoriasis in the Turkish population (Table 6).

## 6. Discussion

The human lymphocyte antigen (HLA) that is located on chromosome 6p21.3 has been associated with both susceptibility and resistance to the development of autoimmune diseases. Studies in different races showed that HLA-C*∗*06 is strongly associated with susceptibility for psoriasis [2–5]. Although the HLA-C*∗*06 allele is widely accepted as a susceptibility gene for psoriasis, the relationship between HLA-C*∗*06 and psoriasis is not definite, as seen with HLA-B*∗*27 and ankylosing spondylitis, and other genes may contribute in the development of psoriasis.

Helms and associates observed that HLA-C alleles have intronic differences and that one difference distinguishes HLA-C*∗*06:02 and HLA-C*∗*12 alleles from all others [7]. According to Helms, HLA-C*∗∗*06 and HLA-C*∗*12 differ by five amino acid replacements. HLA-C*∗*12 alleles are also associated with HLA-B group alleles that are related to psoriasis (HLA-B*∗*38:01 and HLA-B*∗*39:01) in Caucasians [7].

Helms observed in the study that, although HLA-C*∗*06:02 was found in the majority of overtransmitted haplotype harboured by HLA-C*∗*12:03, HLA-C*∗*12 was also associated with HLA-B alleles that play a role in psoriasis (HLA-B*∗*38:01 and HLA-B*∗*901). Mabuchi* et al.* suggested that HLA-C*∗*12:02 and HLA-C*∗*06:02 are allied alleles sharing identical sequences [9]. Mabuchi also found that HLA-C*∗*06 was associated with early-onset psoriasis and that late-onset of psoriasis was significantly associated with HLA-C*∗*12. In contrast, a Brazilian study group found an association between HLA-C*∗*12 and early onset of psoriasis [10]. Recently, Liao and associates stated that HLA-B*∗*27 and HLA-C*∗*12 were associated with psoriatic arthritis in the Chinese population [11].

In our study, we observed that HLA-C*∗*06 is the most frequent allele in patients with psoriasis and was associated with early onset of psoriasis. HLA-C*∗*12 was the second most frequent allele and was associated with PASI scores higher than 10. Those patients carrying HLA-C*∗*12 were also resistant to phototherapy, topical medications, and conventional systemic treatment. It is well documented that obesity, smoking, and alcohol intake play a role in the severity of psoriasis. Unfortunately, we did not investigate those risk factors in patients with high PASI scores in our study group. Evaluation of psoriatic arthritis with CASPAR criteria was done by an experienced rheumatologist. We could not find significant association of arthritis neither with HLA-C*∗*06 nor with HLA-C*∗*12. A multicenter meta-analysis showed that genetic risk factors can differ in different populations. Thus, HLA-C*∗*12 may not be a genetic risk factor universally and can show differences depending on ethnic origin. Our results do not match with Japanese and Brazilian results because we could not observe any association between HLA-C*∗*12 and early or late onset of psoriasis. In contrast with a Chinese study, we did not observe any association between HLA-C*∗*12 and psoriatic arthritis. So, it seems that HLA-C*∗*12 may play role as a genetic susceptibility allele in psoriasis in some populations but associations may show differences. These differences may depend on ethnic origin.

In conclusion, we think that HLA-C*∗*12 is an important susceptibility allele in the Turkish population for psoriasis and may be associated with high PASI scores. Our study is limited because we could not investigate other related genes and risk factors for severe psoriasis.

## Figures and Tables

**Table 1:** The associations between parameters and HLA-C*∗*06.

*Confidence Interval*				
	Minimum	Maximum	Odds Ratio	P-Value
Arthritis	0.3	0.96	0.5	0.038
Age at the time of diagnosis	0.93	0.98	0.95	< 0.001
Psoriasis duration	1.03	1.10	1.06	< 0.001
PASI score	0.96	1.03	1.00	0.805

**Table 2:** PASI scores of HLA-C*∗*12-positive patients were statistically higher than those of HLA-C*∗*12-negative patients.

HLA-C*∗*12	Mean	N	Std. Deviation	Minimum	Maximum	Median
NEGATIVE	8,3989	97	8,07906	,60	40,00	5,4000
POSITIVE	12,8453	53	10,72066	,60	40,00	9,7000
Total	10,0354	150	9,35436	,60	40,00	7,8000

**Table 3:** The associations between parameters and HLA-C*∗*12.

*Confidence Interval*				
	Minimum	Maximum	Odds Ratio	P-Value
Arthritis	0.72	2.75	1.41	0.318
Age at the time of diagnosis	0.99	1.04	1.01	0.417
Psoriasis duration	0.98	1.03	1.00	0.755
PASI score	1.01	1.09	1.05	0.008

**Table 4:** The P result of comparing mean PASI scores of HLA-C*∗*12 positive and negative patients.

Statistics	PASI
Mann-Whitney U	1798,000
Wilcoxon W	5984,000
Z	-2,542
Asym. Sig. (2-tailed)	,011

**Table 5:** The risk of psoriatic arthritis and PASI > 10 scores in patients with HLA-C*∗*12 allele.

	Significance		95% Confidence Interval for HLA-C*∗*12 Positiveness	
			Lower	Upper
Psoriatic Arthritis	0.476	1.268	0.724	2.221
PASI > 10	0.033	1.854	1.006	3.417

**Table 6:** The risk of alleles in psoriatic patients.

*Confidence Interval*				
	Minimum	Maximum	Odds Ratio	P-Value
HLA-C*∗*01	1.15	15.01	4.15	0.030
HLA-C*∗*06	2.49	6.78	4.11	< 0.001
HLA-C*∗*07	0.20	0.59	0.34	< 0.001
HLA-C*∗*12	1.56	4.59	2.68	< 0.001
HLA-C*∗*15	0.1	0.63	0.25	0.004

## Data Availability

The data used to support the findings of this study are available from the corresponding author upon request.
